# Rotavirus Structural Proteins and dsRNA Are Required for the Human Primary Plasmacytoid Dendritic Cell IFNα Response

**DOI:** 10.1371/journal.ppat.1000931

**Published:** 2010-06-03

**Authors:** Emily M. Deal, Maria C. Jaimes, Sue E. Crawford, Mary K. Estes, Harry B. Greenberg

**Affiliations:** 1 Department of Microbiology & Immunology, Stanford University School of Medicine, Stanford, California, United States of America; 2 Department of Medicine, Stanford University School of Medicine, Stanford, California, United States of America; 3 BD Biosciences, San Jose, California, United States of America; 4 Department of Molecular Virology and Microbiology, Baylor College of Medicine, Houston, Texas, United States of America; 5 Veterans Affairs (VA) Palo Alto Health Care System, Palo Alto, California, United States of America; North Carolina State University, United States of America

## Abstract

Rotaviruses are the leading cause of severe dehydrating diarrhea in children worldwide. Rotavirus-induced immune responses, especially the T and B cell responses, have been extensively characterized; however, little is known about innate immune mechanisms involved in the control of rotavirus infection. Although increased levels of systemic type I interferon (IFNα and β) correlate with accelerated resolution of rotavirus disease, multiple rotavirus strains, including rhesus rotavirus (RRV), have been demonstrated to antagonize type I IFN production in a variety of epithelial and fibroblast cell types through several mechanisms, including degradation of multiple interferon regulatory factors by a viral nonstructural protein. This report demonstrates that stimulation of highly purified primary human peripheral plasmacytoid dendritic cells (pDCs) with either live or inactivated RRV induces substantial IFNα production by a subset of pDCs in which RRV does not replicate. Characterization of pDC responses to viral stimulus by flow cytometry and Luminex revealed that RRV replicates in a small subset of human primary pDCs and, in this RRV-permissive small subset, IFNα production is diminished. pDC activation and maturation were observed independently of viral replication and were enhanced in cells in which virus replicates. Production of IFNα by pDCs following RRV exposure required viral dsRNA and surface proteins, but neither viral replication nor activation by trypsin cleavage of VP4. These results demonstrate that a minor subset of purified primary human peripheral pDCs are permissive to RRV infection, and that pDCs retain functionality following RRV stimulus. Additionally, this study demonstrates trypsin-independent infection of primary peripheral cells by rotavirus, which may allow for the establishment of extraintestinal viremia and antigenemia. Importantly, these data provide the first evidence of IFNα induction in primary human pDCs by a dsRNA virus, while simultaneously demonstrating impaired IFNα production in primary human cells in which RRV replicates. Rotavirus infection of primary human pDCs provides a powerful experimental system for the study of mechanisms underlying pDC-mediated innate immunity to viral infection and reveals a potentially novel dsRNA-dependent pathway of IFNα induction.

## Author Summary

Rotaviruses cause severe dehydrating diarrhea and are a leading cause of death in children worldwide. A potent antiviral, interferon-α (IFNα), is rapidly secreted by plasmacytoid dendritic cells (pDCs) in response to viral single-stranded RNA or DNA genomes. Here, we examined the effects of rotavirus on pDCs purified from human blood. We found that very few pDCs supported rotavirus replication, and that pDCs retained similar functionality in response to live or inactivated rotaviruses. While pDCs produced large quantities of IFNα shortly after rotavirus exposure, this was impaired in cells supporting viral replication. We also found that two viral proteins and the rotavirus double-stranded RNA genome were required for the initiation of the pDC IFNα response to rotavirus. Additionally, we found that cleavage of one of these viral proteins, a traditional prerequisite for rotavirus infection in other cell types, was not required for the infection of pDCs or production of IFNα. This may enable the host to rapidly initiate an immune response to rotavirus that subsequently restricts infection to the intestine and contributes to the resolution of disease. Our study provides novel insight into the interaction between rotavirus and the host innate immune response, and also identifies a unique mechanism for the production of IFNα by pDCs.

## Introduction

Dendritic cells (DCs), a highly specialized subset of professional antigen-presenting cells, play a central role in the initiation of innate and adaptive immunity. There are two known major subsets of primary human and murine circulating DCs: myeloid DCs, which function principally in antigen presentation, and plasmacytoid DCs (pDCs), which secrete the type I interferons (IFN), IFNα and IFNβ [1], as well as a variety of other cytokines and chemokines. Viral induction of type I IFN expression has been well studied in recent years and has been shown to be mediated by multiple pattern recognition receptors (PRRs), including retinoic acid-inducible gene (RIG)-I, melanoma differentiation-associated gene (MDA)5, toll-like receptor (TLR)3, TLR7 and TLR9. PRR expression is restricted in pDCs, with only TLR7 and TLR9 implicated in viral-induced IFNα production through the recognition of single-stranded (ss)RNA or DNA, respectively [2], [3], [4], [5], [6], [7], [8], [9]. IFNα production by pDCs is also observed following TLR7/8 stimulus with synthetic resiquimods and imiquimods, or TLR9 antagonism by CpG oligodeoxynucleotides (ODN). pDCs are not generally thought to be able to respond to double-stranded (ds)RNA, as stimulation with poly I:C or long (500 base pairs) dsRNA molecules fail to elicit IFNα or upregulate activation or maturation markers, such as CD86 and CD83 [10], [11], [12]. However, low levels of IFNα production have been reported following pDC stimulation with poly A:U [13] or *in vitro* transcribed viral dsRNA [11], and a recent report indicates a role for RIG-I-like helicases in recognizing replicating virus in murine pDCs lacking the IFN receptor [14]. Short interfering dsRNAs have also been demonstrated to elicit an IFNα response through TLR7, although this appears dependent on a specific sequence, and not on the dsRNA structure [12].

It is well understood that pDCs activate natural killer cells [15], macrophages [15], [16] and T and B cells [17], [18], presumably, in part, through IFNα stimulus. Recent evidence suggests that pDCs may have additional antiviral effects, including the direct inhibition of viral replication in target cells secondary to the secretion of type I IFN [19], [20], [21]. pDCs are also implicated in restricting viral replication *in vivo*, as there is an inverse correlation between circulating pDC numbers and human immunodeficiency virus (HIV) or hepatitis C virus (HCV) viral load [22], [23], [24], [25].

Rotavirus, a dsRNA icosahedral virus in the *Reoviridae* family, is the leading cause of severe dehydrating diarrhea in young children worldwide, with 500,000 to 600,000 annual deaths attributed to rotavirus infections [26], [27], [28], [29]. Significant morbidity and economic impact are the main effects in the United States, accounting for approximately 50,000 to 60,000 hospitalizations a year and loss of time from work for caregivers [30].

Rotaviruses are characterized by a triple-layered protein capsid composed of four major structural proteins. Viral protein (VP)2 comprises the innermost layer, in which the dsRNA genome is contained, while the middle layer consists of VP6. The outer layer of the virion is composed of the VP7 glycoprotein and protease-sensitive VP4 spikes [31]. Although both triple- and double-layered particles (TLPs and DLPs, respectively) are generated during rotavirus replication, only TLPs are infectious, due to the requirements of VP4 and VP7 for cell binding, and trypsin cleavage of VP4 for viral entry and infectivity [32], [33], [34], [35], [36]. Formation of noninfectious empty TLPs and DLPs, which lack the viral genome, is also observed during infection. The nonstructural proteins (NSP1 through 5) are involved in viral replication, morphogenesis and assembly, but they are not expressed by nonreplicating virus and are not part of the infectious virion [31].

Rotavirus-induced immune responses, especially the T and B cell responses, have been extensively characterized; however, little is known about innate immune mechanisms required to control rotavirus infection. Rotavirus structural and nonstructural proteins have been detected in primary DCs from murine mesenteric lymph nodes and spleens [37], [38], and murine bone marrow-derived DCs have been shown to secrete type I IFN and tumor necrosis factor (TNF)α following rhesus (RRV) or bovine rotavirus (RF-81) challenge, respectively [39], [40]. Mature human monocyte-derived myeloid DCs (moDCs) have been demonstrated to be more susceptible to rotavirus infection than immature moDCs, although infection did not result in substantial cell death [41]. Additionally, infection did not induce moDC maturation, but instead promoted priming of Th1 cells [41]. A recent study of total human peripheral blood mononuclear cells (PBMCs) exposed to RRV or human rotavirus indicated that both myeloid DCs and pDCs are susceptible to infection, and that infection results in the secretion of IFNα, presumably from pDCs [42]. While increased levels of IFNα have also been correlated with a positive clinical outcome in infected children [43], [44], several rotaviruses, including RRV, have recently been demonstrated to antagonize the production of type I IFN through the degradation of interferon regulatory factors (IRF)3, IRF5, and IRF7, and the inhibition of NFκB activation, by a viral nonstructural protein, NSP1 [45], [46], [47], [48].

Here we report the effects of rotavirus infection on highly purified primary human pDCs. We demonstrate that although rotavirus initiates detectable transcription and translation in only a small percentage of pDCs, a significant percentage of pDCs activate and mature following exposure to virus. Importantly, we demonstrate that stimulation of pDCs with live or inactivated RRV effects secretion of IFNα and multiple proinflammatory cytokines and chemokines. This response is dependent on the presence of the viral dsRNA genome. As IFNα production by pDCs is classically triggered in response to ssRNA or DNA, the induction of this response by a replication-incompetent dsRNA virus indicates a potentially novel mechanism of viral sensing by pDCs.

## Results

### A subpopulation of human primary peripheral pDCs is permissive to RRV replication

**Figure 1:**
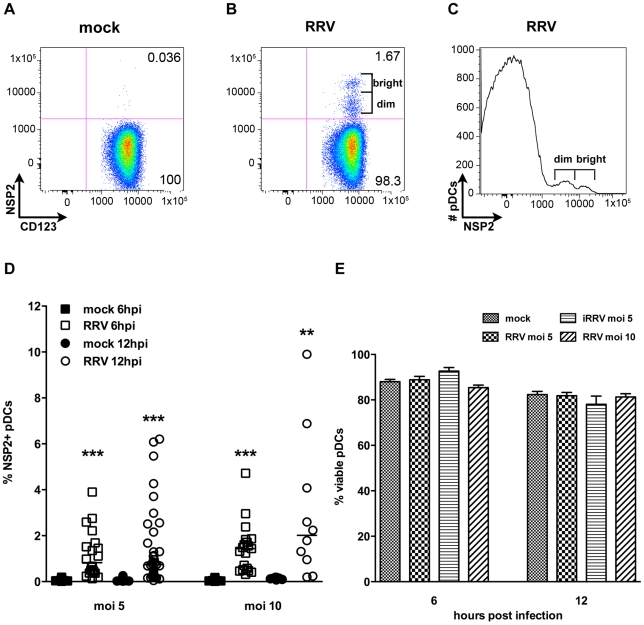
Infection of human peripheral pDCs with rhesus rotavirus. Replication of rhesus rotavirus (RRV) in pDCs was assessed by intracellular staining for NSP2. (A, B) Representative FACS plots of purified pDCs exposed to MA104 supernatant alone (A) or live RRV (moi 10, B) 6 hpi. (C) Representative histogram demonstrating NSP2^dim^ and NSP2^bright^ populations. (D) Percent NSP2^+^ pDCs from multiple healthy donors (n = 9–37) exposed to mock or RRV (moi 5 or 10) stimulus at 6 or 12 hpi. **: p = 0.0039; ***: p≤0.0002 by Wilcoxon signed rank test. (E) Percent viable pDCs 6 or 12 h following mock or rotavirus stimulus. p≥0.125 vs. mock; Wilcoxon signed rank test.

To characterize the interaction between pDCs (phenotypically defined as viable lineage^-^HLA-DR^+^CD11c^-^CD123^+^ cells, Figure S1) and rotavirus, pDCs were purified from human blood by negative selection (mean purity ± standard error mean [SEM]: 86.48%±0.92) and exposed to RRV at a multiplicity of infection (moi) of 5 or 10, or to equivalent quantities of inactivated RRV (iRRV). Expression of NSP2, a viral nonstructural protein expressed only in cells in which rotavirus replicates, was detected by intracellular flow cytometry at 6 and 12 hours post infection (hpi). As illustrated in Figure 1, NSP2 was not detected in pDCs receiving MA104 supernatant (mock-treated; Figure 1A) or iRRV (data not shown), but was expressed in a small but significant percentage (mean ± SEM, moi 5: 1.18%±0.23 6hpi, 1.43%±0.28 12hpi; moi 10: 1.45%±0.25 6hpi, 3.02%±0.99 12hpi) of pDCs inoculated with live RRV (Figure 1B and 1D). Additionally, NSP2 was detected only after pDCs were permeabilized (data not shown). Two populations of NSP2^+^ cells, NSP2^dim^ and NSP2^bright^, were frequently apparent in pDCs exposed to live RRV (Figure 1B and 1C); approximately 20% of donors had only a single NSP2^dim^ population. While the total percent of pDCs expressing NSP2 following RRV challenge increased from 6 to 12hpi, this increase was not significant (moi 5: p = 0.77; moi 10: p = 0.30; Mann-Whitney) (Figure 1D). The percent of NSP2^+^ pDCs also increased with moi (6hpi: p = 0.26; 12hpi: p = 0.063; Mann-Whitney), but remained a minor proportion of the total pDC population (median: 2.01%, moi 10, 12hpi) (Figure 1D). Further increasing the moi to 25 or 100 modestly increased the NSP2^+^ population, but it remained relatively small (mean: 4.99%, median: 2.07%, moi 100, n = 4, data not shown). pDCs from multiple donors (n = 5, mean purity: 91.51%) were titered after overnight culture to determine whether rotavirus infection was productive. A significant increase in viral titer (approximately 20-fold, p≤0.03; Wilcoxon signed rank test) (data not shown) was observed in two of these donors, while the other three showed no significant change over time (p≥0.17; Wilcoxon signed rank test) (data not shown). While pDC viability was significantly decreased from 6 to 12hpi in cultures receiving mock, RRV (moi 5) or iRRV stimulus (p≤0.004; Mann-Whitney), viral exposure did not result in significant cell death compared to mock stimulus at a given time point (p>0.063; Wilcoxon signed rank test) (Figure 1E). The low frequency of NSP2^+^ pDCs shows that the majority of human pDCs are resistant to RRV replication, as defined by evidence of expression of a non-structural rotavirus protein.

### pDCs activate and mature following RRV stimulation

**Figure 2:**
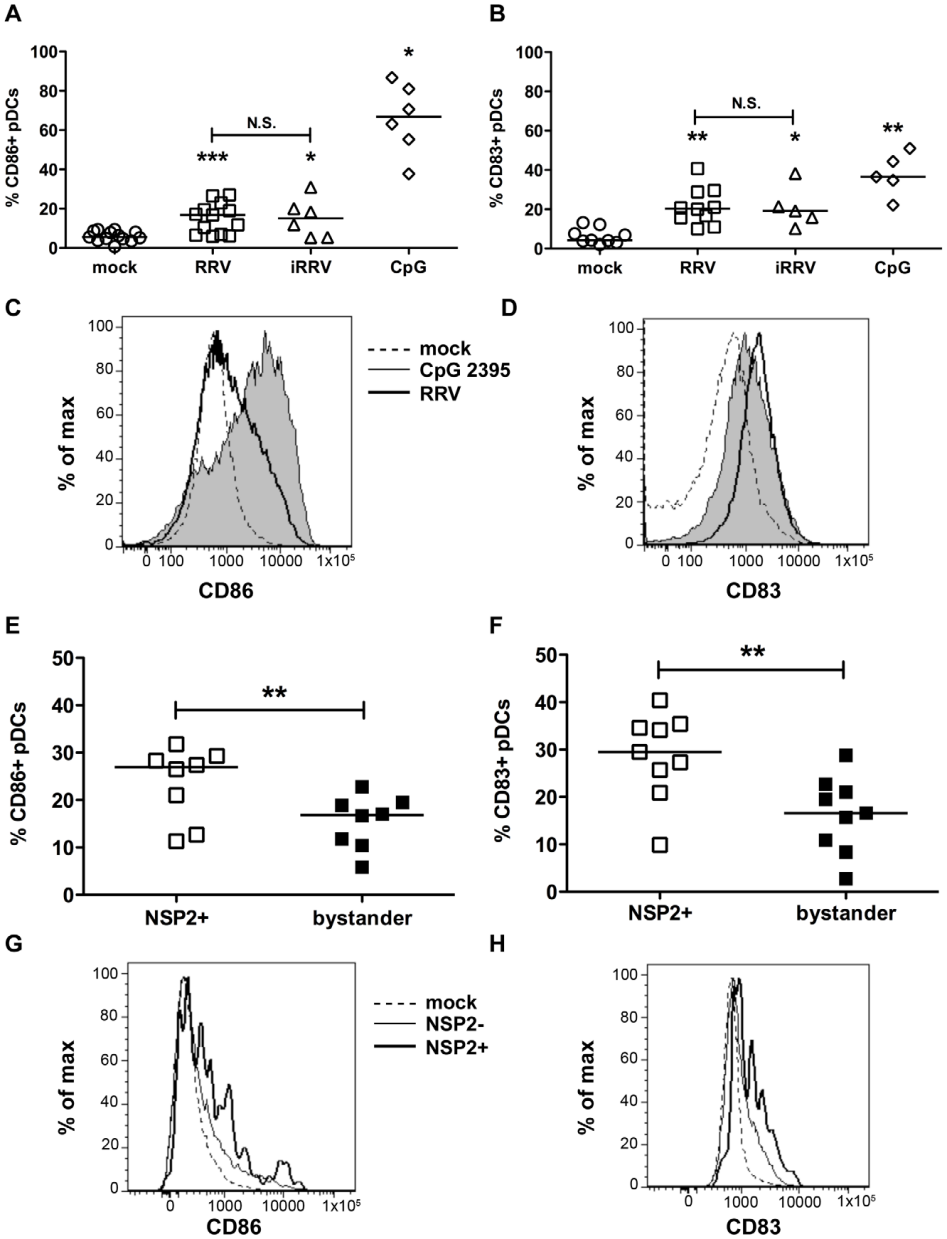
Peripheral pDC activation and maturation following exposure to live or inactivated RRV. The ability of live or inactivated RRV (moi 5), or CpG 2395 (4 µg/ml), to stimulate pDCs was assessed by flow cytometry for expression of activation (A, C, CD86) or maturation (B, D, CD83) markers 12h following stimulus. (A, B) Summary of multiple donors (n = 5–13). *: p = 0.0313; ***: p≤0.0005; Wilcoxon signed rank test. (C, D) Representative histograms showing pDC activation (C) or maturation (D) following exposure to MA104 supernatant alone (dotted line), CpG 2395 (4 µg/ml; shaded) or RRV (moi 5; bold line). (E-H) To determine whether viral replication affected the pDC response, cells exposed to live RRV were gated based on NSP2 expression. The expression of CD86 (E, G) and CD83 (F, H) by NSP2^+^ and bystander (NSP2^-^) pDCs was subsequently assessed. (E, F) Summary of percent CD86^+^ (E) or CD83^+^ (F) pDCs in NSP2^+^ or bystander pDC populations from multiple donors 12hpi (n = 8–9); p≤0.0078; Wilcoxon signed rank test. (G, H) Representative histograms showing pDC activation (G) or maturation (H) of NSP2^+^ (bold) or NSP2^-^ bystander (thin line) pDCs in cultures exposed to RRV (moi 5) or mock stimulus (dotted line).

To establish whether pDCs activate or mature following inoculation with RRV, the frequency of pDCs expressing markers for activation (CD86) or maturation (CD83) was determined by flow cytometry. pDC activation (Figure 2A and 2C) and maturation (Figure 2B and 2D) occurred at similar levels 12h after stimulation with either live or inactivated rotavirus (p≥0.125; Wilcoxon signed rank test), and were significantly increased compared to mock-stimulated cells (p≤0.03; Wilcoxon signed rank test). Stimulation with CpG ODN 2395 resulted in activation of a significantly greater percentage of the pDC population than that observed in pDCs exposed to RRV (p = 0.03; Wilcoxon signed rank test). The percent of pDCs matured by CpG ODN 2395 stimulus was also increased, but not significantly compared to those exposed to RRV (p = 0.094; Wilcoxon signed rank test). Viral replication did not inhibit maturation or activation, as pDCs positive for NSP2 expressed CD86 and CD83 at a significantly higher frequency (p≤0.008; Wilcoxon signed rank test) than bystander pDCs (Figure 2E-H). Expression of both molecules was significantly increased in both populations compared to pDCs receiving mock stimulus. Together, these data demonstrate that pDCs retain the ability to activate and mature after viral exposure, likely due to secondary effects, as the presence of replicative rotavirus is dispensable for induction of these phenotypes.

### Cytokine production by human primary pDCs in response to rotavirus is independent of viral replication

**Figure 3:**
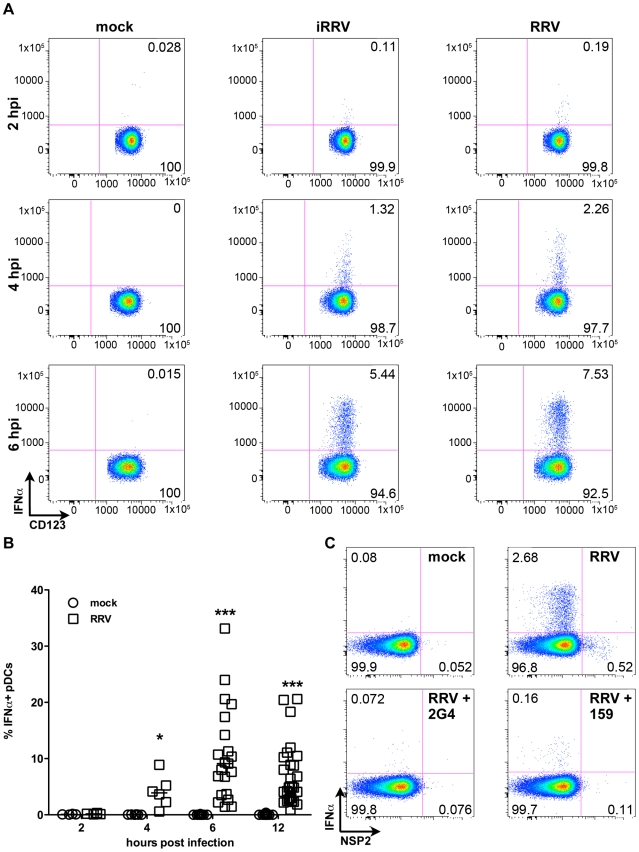
Intracellular production of IFNα by pDCs after exposure to live or inactivated RRV. (A) Representative FACS plots of IFNα vs. CD123 expression by pDCs exposed to mock, iRRV or RRV stimulus 2-6hpi (moi 5). (B) Time course of IFNα induction in pDCs after mock or RRV (moi 5) stimulus (n = 3–30). *: p = 0.0313; ***: p≤0.0002; Wilcoxon signed rank test. (C) IFNα induction and NSP2 production in pDCs exposed to RRV in the presence or absence of neutralizing VP4 (2G4) or VP7 (159) mAb.

**Figure 4:**
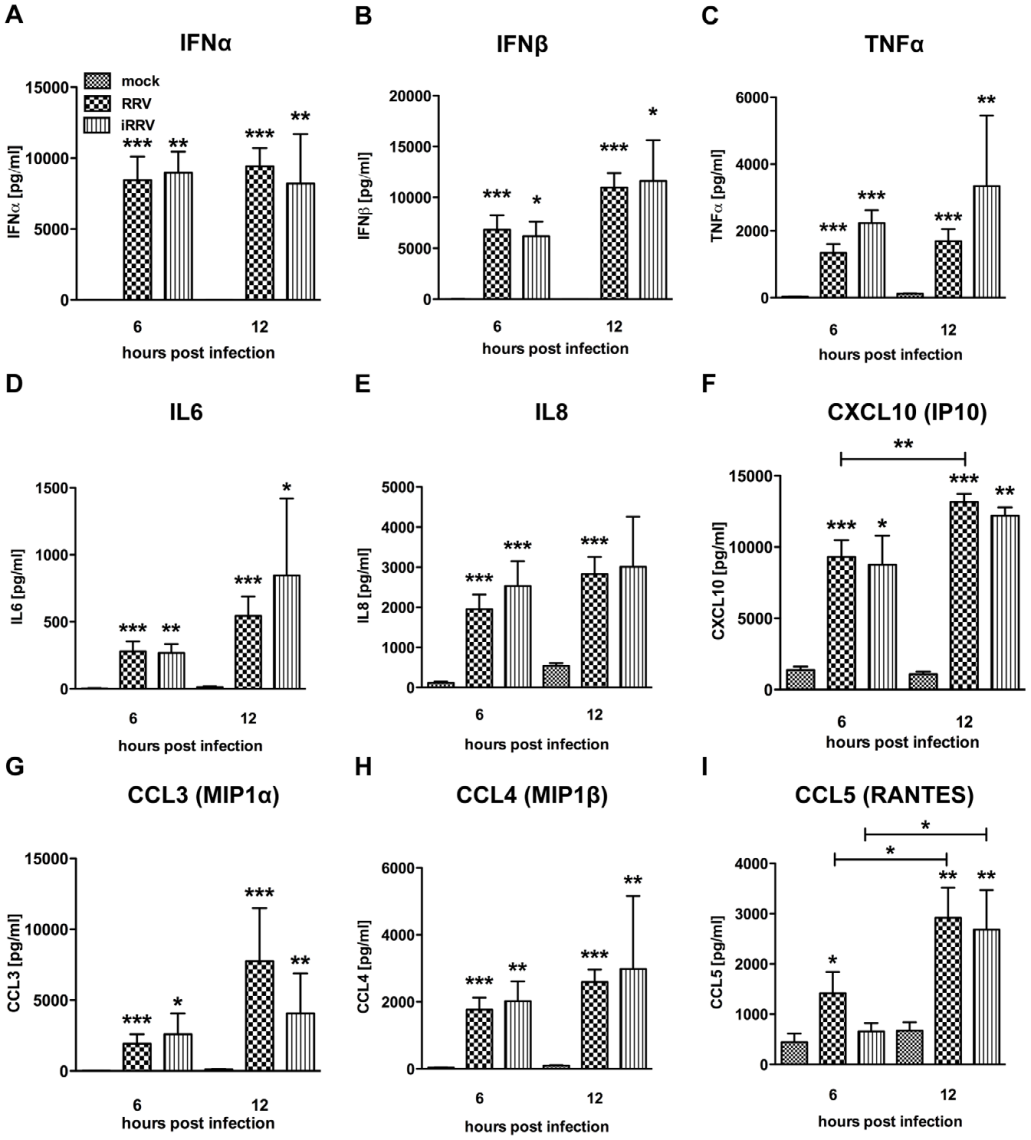
Cytokine secretion by pDCs following exposure to live or inactivated RRV. Detection of secreted IFNα (A), IFNβ (B), TNFα (C), IL6 (D), IL8 (E), CXCL10 (IP10) (F), CCL3 (MIP1α) (G), CCL4 (MIP1β) (H), and CCL5 (RANTES) (I) in culture supernatants (n = 3–18) from pDCs (1 million per mL) exposed to mock, live or inactivated RRV (moi 5) stimulus at various times post infection by Luminex or ELISA. TNFβ (LTA): ≤100 pg/mL detected post viral stimulus. sCD40L: no observed increase versus mock stimulus. Not detected: IFNγ, IL10, IL12p40, IL12p70, IL1β, IL1RA, IL2 and IL4. *: p≤0.0265; **: p≤0.0091; ***: p≤0.0008; Mann-Whitney.

Production of IFNα is an important component of the pDC-derived antiviral response. To assess this component of the pDC anti-rotaviral response, the frequency of cells expressing IFNα was determined by flow cytometry. Intracellular IFNα was detected within 4hpi of RRV stimulus, peaked at 6hpi and was sustained until at least 12hpi (Figure 3A and 3B). IFNα was detected intracellularly at similar frequencies in pDCs exposed to iRRV (which was both infectivity and transcriptionally negative) (p = 0.815; Mann-Whitney) (Figure 3A and data not shown), suggesting that replication or transcription of rotavirus is largely dispensable for IFNα induction in human pDCs. Intracellular IFNα was abrogated by the addition of RRV neutralizing VP7 or VP4 monoclonal antibodies (either mAb 159 or 2G4), indicating that the observed induction was virus-specific and likely dependent on an event occurring after virus binding and entry (Figure 3C) [49]. To identify and quantify IFNα secretion, and to detect other cytokines and chemokines secreted in response to RRV exposure, Luminex assays or ELISAs were performed on supernatants collected from inoculated pDCs at various times post infection. Only supernatants from pDC cultures ≥85% pure (mean: 90.84%±0.75; n = 3–18) were analyzed to minimize the contribution of contaminating cells to the cytokine milieu. A significant IFNα response, approaching 10,000 pg IFNα/ml, was detectable 6h following exposure to live or inactivated RRV (Figure 4A). In addition, significant quantities of IFNβ, TNFα, interleukin (IL)6, IL8, CXCL10 (IP10), CCL3 (MIP1α), CCL4 (MIP1β), CCL5 (RANTES) and TNFβ were detected at various times post infection (Figure 4B-I and data not shown). Together, these data suggest that live or inactivated RRV rapidly induce production of significant levels of IFNα and other proinflammatory cytokines and chemokines by pDCs.

### IFNα production is impaired in pDCs undergoing RRV replication

**Figure 5:**
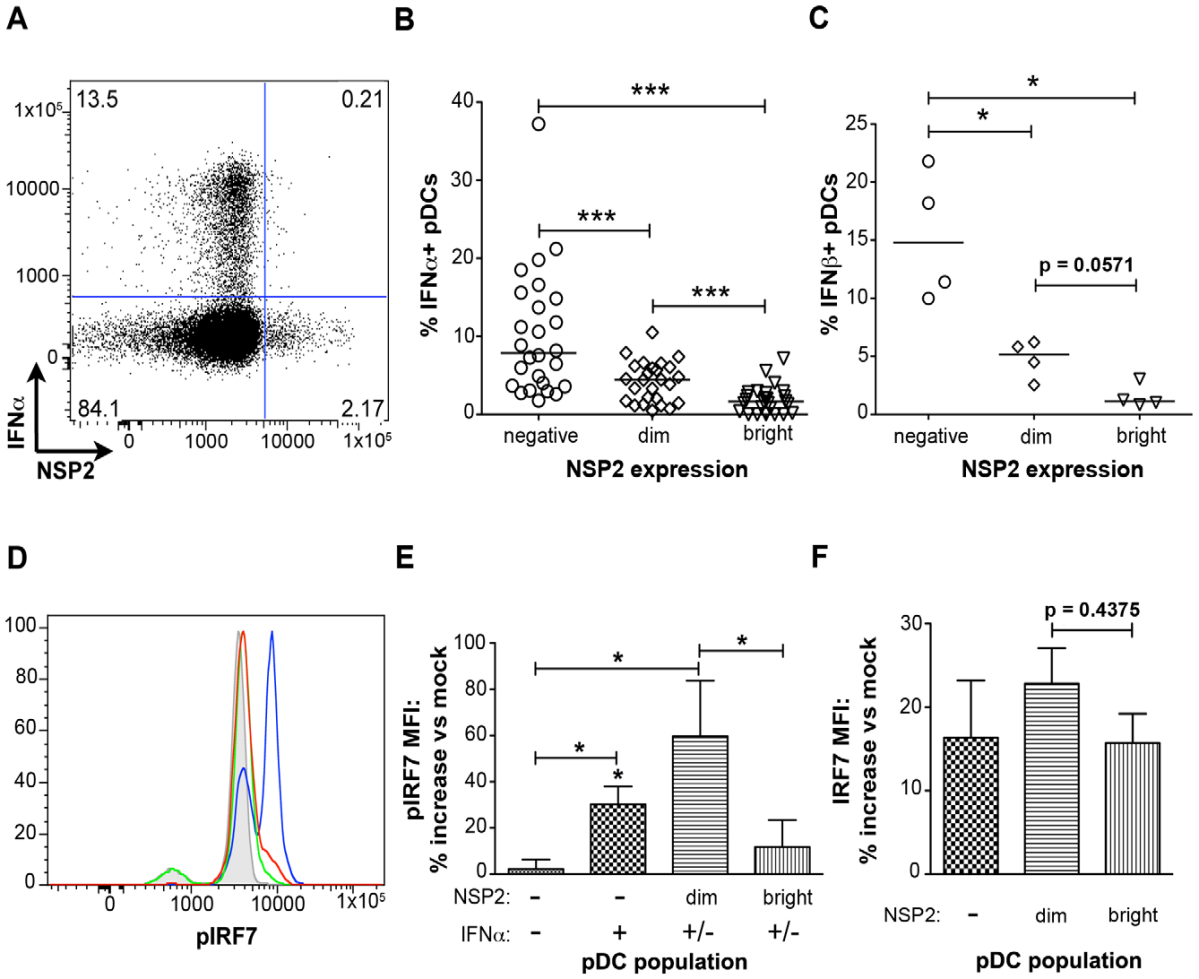
The effect of viral replication on IFNα induction and IRF7 phosphorylation. (A) Representative FACS plot of IFNα vs. NSP2 expression in pDCs stimulated with RRV 6hpi (moi 10). (B, C) pDCs from multiple donors were exposed to RRV and the percent of IFNα^+^ (B) (n = 26) or IFNβ^+^ (C) (n = 4) pDCs negative, dim or bright for NSP2 were determined. *:p = 0.0286, ***: p≤0.0006; Wilcoxon signed rank test. Donors with fewer than 0.5% of pDCs staining positive for NSP2 were excluded from this analysis. (D) Representative histograms of IRF7 phosphorylation in NSP2^bright^ (red), NSP2^dim^ (blue), and NSP2^-^ (green) pDCs 6 hours post RRV or mock (gray) stimulus. (E, F) Percent increase in pIRF7 (E) or total IRF7 (F) MFI by indicated pDC populations relative to mock treated pDCs from multiple donors (n = 6), 6 hours post RRV inoculation. *: p = 0.0313; Wilcoxon signed rank test.

In contrast to what was observed with pDC activation and maturation, IFNα production was significantly reduced in NSP2^+^ pDCs (Figure 5). NSP2^-^ bystander pDCs produced IFNα at a significantly increased frequency (p<0.0001; Wilcoxon signed rank test) compared to pDCs dim or bright for NSP2 (Figure 5A and 5B). The percent of IFNα^+^ pDCs was significantly increased in the NSP2^dim^ population compared to that in the NSP2^bright^ (p = 0.0006; Wilcoxon signed rank test), supporting the hypothesis that increased levels of RRV infection are inversely correlated with IFNα production. More significant was the sustained production of IFNα in uninfected (NSP2^-^) pDCs following RRV stimulus. A similar correlation between NSP2 expression and IFNβ production was also observed (Figure 5C). These data indicate that infection in a minor subset of pDCs results in the local suppression of the IFNα response in these specific cells.

### IRF7 is phosphorylated in pDCs supporting low levels of viral replication

To further investigate the impairment of the IFNα response in pDCs undergoing rotavirus replication, phosphorylation of IRF7, the key transcription factor for the IFNα response in pDCs [50], was assessed by phosflow (Figure 5D and 5E). In accordance with the decreased production of IFNα by NSP2^bright^ cells compared to that of NSP2^dim^ pDCs, as well as previous findings of IRF7 degradation in rotavirus-infected cells [45], [46], phosphorylated IRF7 (pIRF7) was substantially decreased in pDCs bright for NSP2 (p = 0.03 versus NSP2^dim^ pDCs; Wilcoxon signed rank test) (Figure 5D and 5E), and is similar to that of IFNα^-^NSP2^-^ pDCs (p = 0.56; Wilcoxon signed rank test) (Figure 5E). To determine whether this decrease in pIRF7 was due to decreased levels of total IRF7 in pDCs, the MFI of total IRF7 was assessed (Figure 5F). While the total IRF7 MFI was slightly depressed in NSP2^bright^ compared to NSP2^dim^ pDCs, this decrease was not significant (p = 0.44; Wilcoxon signed rank test) and thus does not account for the observed differences in IRF7 phosphorylation. The increased levels of pIRF7 in NSP2^dim^ pDCs may represent continual, direct stimulus of the IRF7 pathway in virus-positive pDCs, as well as a mechanism by which the IFNα response is directly initiated. Alternately, pIRF7 may be sequestered in a nontraditional cellular compartment prior to degradation, leading to increased protein accumulation. Interestingly, levels of IRF7 phosphorylation in IFNα^+^NSP2^-^ pDCs were intermediate to that of NSP2^dim^ (p = 0.31; Wilcoxon signed rank test) and NSP2^bright^ or NSP2^-^IFNα^-^ pDCs (p = 0.0313; Wilcoxon signed rank test) (Figure 5E). The slight depression of pIRF7 in the IFNα^+^ population compared to that observed in NSP2^dim^ pDCs may be attributable to the enhanced IFNα production in this subpopulation. As pIRF7 is degraded following the initiation of IFNα transcription, cells in which IFNα has been transcribed would be expected to have lower levels of pIRF7 than those negative for IFNα. Notably, inhibition of IFNα secretion, achieved by simultaneous addition of brefeldin A with viral inoculation, decreased the percent of IFNα^+^ pDCs by an average of 63.5% (n = 6, data not shown). This supports the notion that recognition of secreted IFNα is an essential component of the IFNα response following rotavirus exposure, as has been reported with Newcastle disease virus [14]. Importantly, this is the first demonstration of impaired IRF phosphorylation in primary human cells infected with rotavirus.

### Trypsin cleavage of VP4 is dispensable for pDC infection and IFNα induction

**Figure 6:**
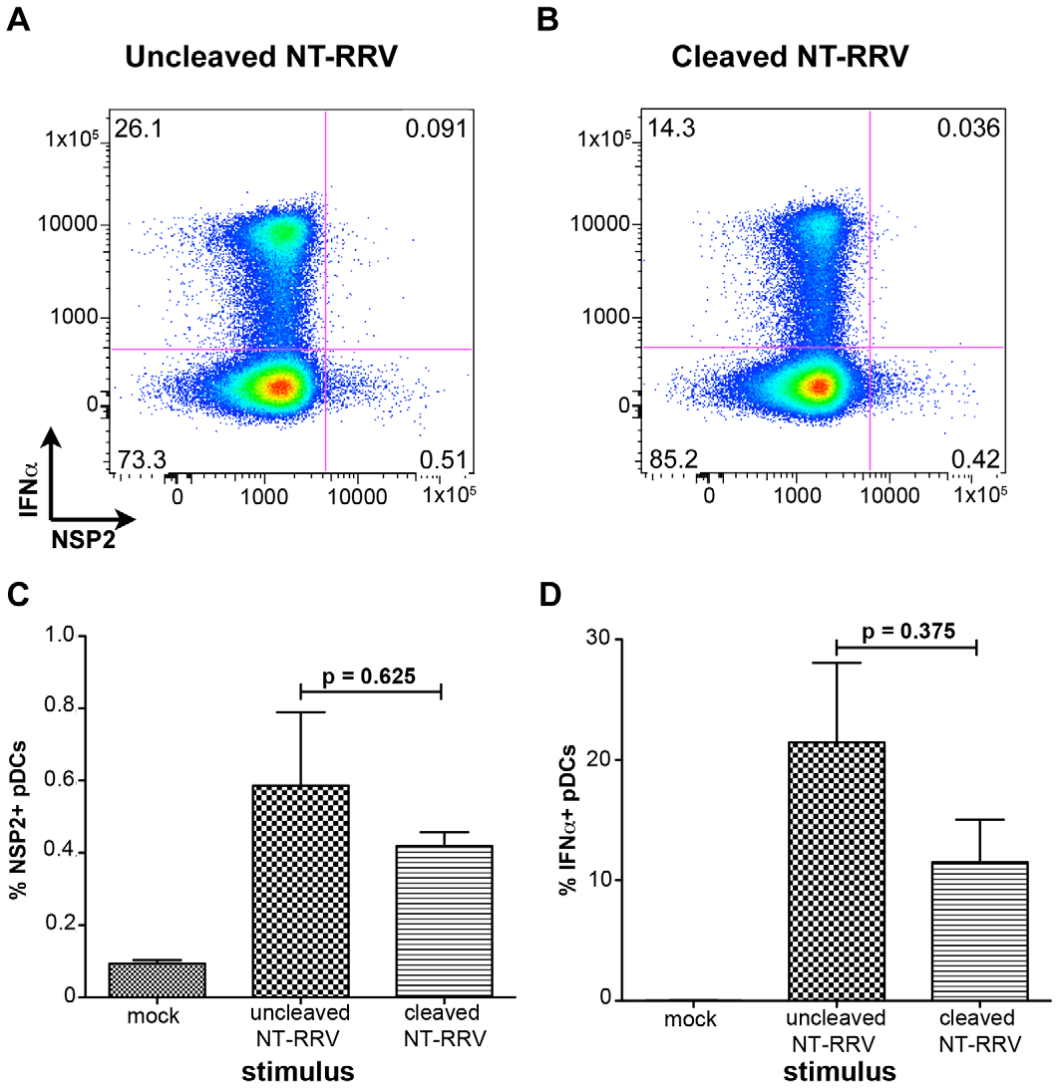
The effect of trypsin activation of rotavirus on pDC infection and IFNα induction. (A, B) Representative FACS plots of IFNα and NSP2 expression by pDCs exposed to equal quantities of non-trypsinized RRV (NT-RRV) with uncleaved (A, 3.77×10^5^ focus forming units/ml; moi = 0.079) or cleaved (B, 2.38×10^7^ focus forming units/ml; moi = 5) VP4. (C, D) Summary of percent NSP2^+^ and IFNα^+^ pDCs from multiple donors (n = 3) following exposure to cleaved or uncleaved RRV. p≥0.375; Wilcoxon signed rank test.

Trypsin-mediated cleavage of VP4 is canonically required for productive entry of rotavirus into permissive cells [33], [34], [35], [36]. However, it has previously been reported that murine bone marrow-derived DCs activate following exposure to uncleaved bovine rotavirus [40], indicating that VP4 cleavage may be dispensable for the induction of the DC response. To investigate the role of trypsin-mediated cleavage of VP4 in both rotavirus infection of, and IFNα production by, human pDCs, we exposed pDCs to RRV grown in the absence of trypsin (non-trypsinized; NT-RRV). Although subsequent exposure to trypsin (thus cleaving VP4) increased infectivity of the uncleaved NT-RRV preparation 63-fold by plaque titration and intracellular NSP2 staining by 20-fold in MA104s (data not shown), examination of NSP2 staining showed that similar proportions of pDCs were infected with either the cleaved or uncleaved NT-RRV preparations (p = 0.625; Wilcoxon signed rank test) (Figure 6A-C). Likewise, uncleaved NT-RRV induced IFNα production by a similar (p = 0.375; Wilcoxon signed rank test) percentage of pDCs, compared to cleaved NT-RRV (Figure 6A, 6B and 6D). Thus, cleavage of VP4 by trypsin is dispensable for both pDC infection and IFNα induction by RRV.

### IFNα induction requires VP4 and/or VP7, and dsRNA

**Figure 7:**
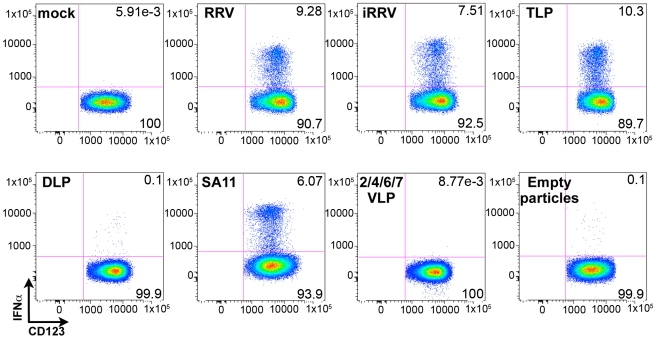
Viral determinants of IFNα induction. Representative FACS plots of IFNα production by pDCs at 12 hours post exposure to rotavirus-derived stimuli. RRV: live virus; iRRV: inactivated virus; TLP: purified triple-layered (2/4/6/7) genome-containing virus particles; DLP: purified double-layered (2/6) genome-containing virus particles; SA11: live virus strain from which VLPs are derived; 2/4/6/7 VLPs: recombinant genome-free virus-like particles; empty particles: purified genome-free TLPs.

Although the ability of pDCs to mount an IFNα response to viral infection is well described, the only documented viral TLR ligands for pDCs are ssRNA and DNA [2], [3], [7], [8], [9]. As rotavirus is a dsRNA virus and since psoralen-treated, transcriptionally-inactive rotavirus efficiently induced IFNα (Figures 3 and 4A), we investigated the viral requirements for IFNα induction. pDCs stimulated with equal quantities (see Methods describing particle quantification) of purified TLPs, DLPs, empty particles, or genome-free recombinant virus-like particles (VLPs) were stained for intracellular IFNα. As expected, density gradient purified RRV TLPs stimulated similar levels (p = 0.695; Wilcoxon signed rank test; n = 10) of IFNα production compared to pDCs treated with unpurified live or inactivated RRV expressing equivalent amounts of VP4, as measured by hemagglutination titer (Figure 7). pDCs exposed to 2/6 genome-containing RRV DLPs expressed neither IFNα nor NSP2, indicating a likely dependence on VP4 or VP7 for viral entry, as previously reported [32]. Alternately, one or both of these viral proteins could be serving as the pathogen-associated molecular pattern (PAMP) responsible for inducing the pDC IFNα response. To address this, pDCs were stimulated with 2/4/6/7 VLPs or 2/6/7 green fluorescent protein (GFP)-VLPs. Both 2/6/7 GFP-VLPs and 2/4/6/7 VLPs were capable of entering pDCs, but failed to induce IFNα (Figure 7 and data not shown). 2/6 VLPs were comparatively defective in the ability to enter pDCs (data not shown), thus supporting the hypothesis that DLPs fail to induce IFNα due to inefficient viral entry. The ability of infectious SA11, the parental virus from which the 2/4/6/7 VLPs were derived, to induce IFNα production (Figure 7) suggests that the lack of IFNα following inoculation with 2/4/6/7 VLPs was due to the absence of a viral genome, and not to strain variation in surface proteins. Consistent with this hypothesis, IFNα was detected in very few pDCs exposed to empty TLPs, as compared to pDCs exposed to equal quantities of genome-containing TLPs. Instead, IFNα production by pDCs exposed to empty particles was consistent with that observed in pDCs inoculated with an equal infectious dose of TLPs, suggesting that residual contaminating viral genome was responsible for IFNα production by the empty TLPs. Together, these data indicate that viral entry, mediated by VP4 and/or VP7, and the viral dsRNA genome are likely required for rotavirus-induced IFNα production by pDCs.

### Endosomal acidification is required for initiation of the pDC IFNα response to rotavirus

**Figure 8:**
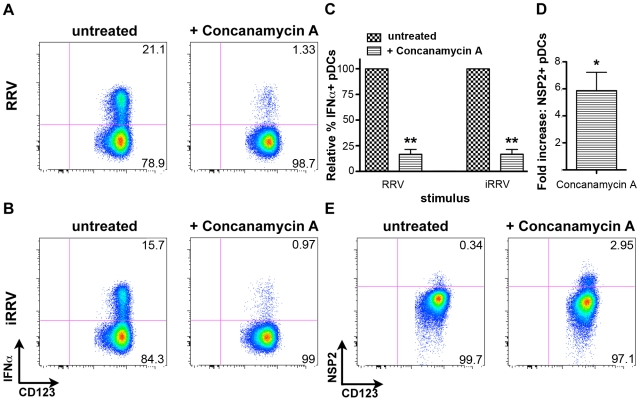
The effects of concanamycin A on IFNα induction and NSP2 expression. Intracellular IFNα was assessed in untreated or concanamycin A-treated pDCs following exposure to RRV (A) or iRRV (B). (C) Relative frequency of IFNα^+^ pDCs in untreated or concanamycin A-treated pDCs from multiple donors (n = 6) following exposure to live or inactivated rotavirus. **: p = 0.0097; Mann-Whitney. (D) Fold increase in NSP2 expression by pDCs from multiple donors (n = 6) treated with concanamycin A prior to infection, compared to expression by untreated pDCs. *: p = 0.0313; Wilcoxon signed rank test. (E) Representative FACS plots depicting NSP2 staining in untreated and concanamycin A-treated pDCs following RRV exposure.

To begin to characterize the mechanism by which pDC recognition and subsequent induction of the IFNα response occurs following rotavirus exposure, pDCs were treated with concanamycin A, a potent inhibitor of endosomal acidification, and thus TLR7/9 function [9], [51], [52], prior to inoculation with either live or inactivated RRV at an moi of 10 (Figure 8A–8C). Subsequent examination of intracellular IFNα production revealed significant inhibition in concanamycin-treated pDCs compared to untreated cells (mean ± SEM, RRV: 83.3%±4.6; iRRV: 83.2%±4.5; p = 0.01; Mann-Whitney). To determine whether the observed lack of IFNα production was due to a defect in viral entry, concanamycin-treated pDCs were examined for NSP2 expression. Interestingly, NSP2 expression was significantly increased in concanamycin-treated pDCs compared to untreated cells (Figure 8D and 8E) (mean fold increase: 5.9; p = 0.03; Wilcoxon signed rank test; n = 6). These data indicate that concanamycin-induced inhibition of endosomal acidification does not inhibit viral infection of pDCs and hence, presumably, does not restrict viral entry. These data support the conclusion that concanamycin A treatment prevents the pDC IFNα response at the level of ligand recognition, implicating TLR7 or TLR9. Additionally, the data suggest that the observed low levels of viral replication in pDCs (Figure 1D) are due, at least in part, to suppression by the pDC IFN response, as NSP2 expression significantly increases following IFNα inhibition (Figure 8C and 8D). Notably, these experiments demonstrate that endosomal acidification is required for the pDC IFNα response to rotavirus, and provide indirect evidence for the involvement of an endosomal receptor, such as TLR7 or TLR9, in the induction of this response.

## Discussion

pDCs play a vital role in the generation of innate and adaptive immunity following viral infection, primarily through the production of large quantities of IFNα in response to stimulus of TLR7 or TLR9 PRRs by ssRNA or DNA PAMPs, respectively. Traditional receptors for dsRNA, such as RIG-I, MDA5 and TLR3, are largely thought to not play a role in the initiation of the IFN response in human pDCs [2], [3], [4], [5], [6], [7], [8], [9]. Although Newcastle disease virus normally elicits an IFNα response in murine pDCs through triggering TLR7, RIG-I-like helicases have been recently implicated in IFNα induction by replicating virus in murine pDCs lacking the IFN receptor [14]. Hence, the mechanisms governing efficient IFNα induction in these cells by transcriptionally inactivated, replication-incompetent dsRNA rotavirus are not yet understood. As rotaviruses represent a large burden of human disease, it will be important to better understand the immune mechanisms underlying their recognition and subsequent generation of antiviral immunity.

In the present study, we demonstrate that primary human pDCs are largely resistant to rotavirus replication, as intracellular staining for NSP2 was observed in only a small percentage (≤5%) of cells following RRV exposure, even at a non-physiologically relevant moi of 100. While productive infection was observed in a subset of donors, this was significantly less than that observed in cells highly permissive for rotavirus infection [53]. As rotavirus is generally a lytic virus [31], [54], [55], the observed lack of significant death in pDC preparations exposed to live RRV, compared to those receiving mock stimulus or inactivated virus, further indicates that few pDCs are permissive to viral replication. These findings are in agreement with previous reports of low frequencies of pDC infection in virus models such as herpes simplex virus 2 [56], Dengue [57], human cytomegalovirus [17], influenza [58], HCV [24] and HIV [59]. The inability of the primary human peripheral pDC population to be uniformly infected by rotaviruses may be attributable to multiple factors, including age of the individual pDC, stage of the cell cycle, or cell-surface marker expression. Additionally, functionally-distinct pDC subsets have been recently identified on the basis of CD2 expression [60]; studies are currently underway to elucidate the role of these subsets in the pDC response to rotavirus.

Consistent with the general resistance to rotavirus infection, pDCs retained several important functional abilities following RRV exposure, as evidenced by their activation, maturation and cytokine production. Evidence of this functionality was observed primarily in the major NSP2^-^ bystander population**,** although activation and maturation were significantly enhanced in the few NSP2^+^ pDCs, as well. The induction of this phenotype by inactivated RRV further indicates that the pDCs are responding to the presence of input virus or to secreted factors, and not to viral replication. The upregulation of DC costimulatory and maturation markers suggests antigen presentation to T cells is preserved, in line with previous reports that pDCs are necessary for stimulation of IFNγ-secreting memory T cells [42]. The antigen presentation hypothesis is supported by increased expression of CD86 and CD83 on NSP2^+^ pDCs compared to bystander pDCs, indicating that the presence of virus enhances this phenotype. Conversely, IFNα/β production is impaired in NSP2^+^ pDCs. Combined with decreased IRF7 phosphorylation in NSP2^bright^ pDCs, this suggests the direct inhibition of the type I IFN response by rotavirus replication, as has been previously observed in fibroblasts and epithelial cells [45], [46], [47], [48].

This study is the first to demonstrate that rotavirus, a segmented dsRNA virus, directly induces IFNα and IFNβ production by primary human pDCs. While it has previously been reported that exposure to rotavirus induces IFNα secretion by murine FLT3 ligand-driven pDCs [39] and total human PBMCs [42], and that this response required pDCs [42], it was unclear until now whether pDCs produced IFNα directly in response to virus, or if pDCs amplified IFNα production in response to type I IFN or some other signal from a non-pDC. In a limited number of experiments, Mesa *et al* [42] observed not only pDCs singly positive for IFNα or rotavirus, but also cells double-positive for rotavirus and IFNα at a greater frequency than that reported here. It is important to note that Mesa *et al* used NSP4 as a marker for rotavirus infection. Extracellular NSP4 has been identified on uninfected cells [61]. Conversely, NSP2, employed in this report, to date has only been detected intracellularly. As such, the percentage of cells identified as positive for rotavirus on the basis of NSP4 staining could be inflated versus those expressing NSP2, as NSP4 staining may include pDCs not truly supporting viral replication; in turn, the percent of NSP4^+^IFNα^+^ pDCs would be artificially increased. This, combined with differences in sample size, may explain the discrepancies in the incidence of NSP4^+^IFNα^+^ pDCs (n = 3) observed by Mesa *et al* and the frequency of NSP2^+^IFNα^+^ pDCs (n = 27) reported here.

pDCs possess a seemingly unique phenotype in the context of rotavirus infection, as the ability of NSP1 to degrade multiple IRFs appears to subvert the type I IFN response in many other cells, including epithelial and fibroblastic cells [45], [46], [62]. Presumably, the constitutive expression of IRF7 in pDCs [63] allows for rapid induction of IFNα following rotavirus exposure, thus effectively suppressing viral replication—and the production of NSPs—in the majority of pDCs through the establishment of a largely paracrine-induced antiviral state. This conclusion is supported by the observed lack of significant increase in the NSP2^+^ or IFNα^+^ pDC populations when the moi is increased to 25 or 100 (data not shown). Importantly, the decrease in IFNα and pIRF7 in pDCs expressing high levels of NSP2 illustrates that the local suppression of the interferon response by actively replicating virus [45], [46], [47], [48] is conserved in human pDCs at an individual cell level. However, inhibition in this minor population is unlikely to substantially modulate the amount of cytokine produced by the total pDC population. The present study provides the first formal demonstration of type I IFN inhibition in primary human cells in which rotavirus replicates.

The uniqueness of pDCs in the context of rotavirus infection is further demonstrated by the ability of trypsinized and non-trypsinized rotavirus to induce IFNα and NSP2 expression at similar frequencies. The long-standing understanding of the requirement for VP4 proteolytic cleavage by luminal trypsin to activate viral entry and infection in the gut led to the assumption that efficient rotavirus infection could not occur systemically due to absence of appropriate extracellular proteases. Rotavirus infection was thought to be largely constrained to the intestine because this was the only anatomical location with substantial amounts of active extracellular trypsin available. The apparent dispensability of this proteolytic requirement for pDC infection may represent an alternate mechanism of rotavirus entry. This “non-trypsin dependent” mechanism of infectious entry might also account for the low levels of systemic organ infection and spread that normally occurs during rotavirus infection, and the elevated levels seen during rotavirus infection of highly immunocompromised people or animals, where the systemic availability of trypsin to proteolytically activate rotavirus is unlikely. Additionally, this provides a mechanism for the establishment of an antiviral state systemically, where circulating virus would not be expected to be able to infect cells in the traditional, trypsin cleavage-dependent, manner.

We have demonstrated that both live and inactivated rhesus rotavirus efficiently stimulate secretion of type I IFN, in addition to many other cytokines, suggesting that transcription of viral nucleic acid is not required for either viral recognition or subsequent cytokine production by primary human pDCs. Conversely, viral replication is required for activation of the IFN response in fibroblasts [62]. Indeed, further analysis of the viral requirements for IFNα induction suggests that pDCs recognize and respond to input rotavirus dsRNA or some degradation product of the rotavirus genome. This is supported by the inability of genome-free TLPs to effect IFNα secretion, indicating that the lack of IFNα induction following VLP-stimulus is due to the absence of a nucleic acid ligand and not to the lack of a minor structural protein, such as VP1 or VP3. To our knowledge, this is the first direct demonstration of the requirement for native viral dsRNA in the initiation of an IFNα response from primary human pDCs.

Importantly, this finding may represent an alternative mechanism of IFNα induction in primary human pDCs. The ability of a pDC that takes up rotavirus to mount a vigorous type I IFN response independently of rotavirus mRNA or protein expression provides a potential mechanism by which the host could effectively circumvent the anti-IFN effects of NSP1-mediated IRF degradation [62]. At least three possible mechanisms exist for this phenotype: first, input or self nucleic acid released from necrotic or apoptotic cells may serve as PAMPs in neighboring cells, as has been previously described [64]. As RRV exposure, either to replication-competent or inactivated virus, does not induce substantial pDC death (Figure 1), and as the cytokine response occurs rapidly following infection, this scenario seems unlikely. Secondly, input viral dsRNA may be exposed in the cytosol, the traditional cellular location for rotavirus replication, through the process of viral replication and interact with a cytosolic receptor such as RIG-I or protein kinase R (PKR). Rotavirus and reovirus, both members of the *Reoviridae* family, have been demonstrated to induce IFNβ production in epithelial and human embryonic kidney 293T cells, respectively, via RIG-I, but not PKR or TLR3 [65], [66]. The mechanism by which input viral genomic RNA recognition might occur is unclear, however, as viral dsRNA is thought to remain encapsidated in the DLP and not be free in the cytoplasm during viral replication. Finally, rotavirus particles may be taken up by and degraded within the endosome, where dsRNA or its degradation products, including ssRNA fragments or a single strand of the dsRNA [12], would be able to interact with TLR3, TLR7 or TLR9. As a biological role for known dsRNA receptors, such as RIG-I, PKR or TLR3, has not been demonstrated in human pDCs [2], [3], [4], [5], [6], it is unlikely that these receptors are responsible for the observed IFNα induction in this system, although RIG-I like helicases have been recently implicated in the generation of a type I IFN response in pDCs from IFN receptor knockout mice [14]. Signaling by a cytosolic receptor should be abrogated if inhibition of endosomal acidification in turn prevented rotavirus entry into the cytosol. However, the persistence and even increase of NSP2 expression following treatment with concanamycin A indicates that pDC entry by rotavirus is conserved under conditions that alter endosomal pH and decrease the pDC IFN response (Figure 8). Thus, the substantial reduction of the IFNα response following concanamycin A treatment supports the notion that an endosomal receptor, such as TLR7 or TLR9, is involved in the initiation of the pDC response to rotavirus. Hence we predict that dsRNA derived from input virus is exposed following viral degradation, and not replication, as inactivated RRV, but neither genome-free TLPs nor VLPs, is sufficient for pDC activation/maturation and IFNα production. While it is possible that recognition of some combination of VP4, VP7 and dsRNA are required for IFNα induction, we instead believe that viral entry—mediated by VP7 and/or VP4—and subsequent recognition of genomic dsRNA or its degradation products are the critical components of this pathway.

The induction of both IFNα and TNFα by rotavirus may create a synergistic effect in the suppression of viral replication, as has been observed following RIG-I stimulus by myxoma virus [67]. Although the precise kinetics of induction are unclear, both TNFα and IFNα are also observed in pDC supernatants following influenza infection [24], [68], [69]. Interestingly, production of IFNα is delayed until 14–20 h following infection with a laboratory strain of H1N1 influenza, at which point ∼4 ng/mL per million pDCs is detectable; this production was dependent on viral replication [69]. Conversely, we report that pDCs exposed to either live or inactivated rotavirus secrete approximately 10 ng/mL of IFNα by 6hpi. In agreement with previous findings [14], the recognition of secreted IFNα is an important component of the amplification of the pDC IFNα response to both live and inactivated rotavirus. As such, the recognition of input viral genome and subsequent induction of an initial IFNα response prior to possible IRF7 inhibition allows for the establishment of an antiviral state by the global pDC population. While inactivated herpes simplex virus also induces IFNα, replication of vesicular stomatitis virus, Sendai virus and human respiratory syncytial virus has been demonstrated to be required for IFNα induction in pDCs [70], [71]. Together, this demonstration of rapid, potent, replication-independent induction of cytokine by a dsRNA virus, largely dependent on endosomal acidification, points to a novel mechanism leading to production of IFNα by human pDCs.

Peripheral pDCs likely represent a significant source of the systemic IFNα observed following *in vivo* rotavirus infection [43], [72], [73], [74]; the dispensability of proteolytic activation of VP4 for pDC infection and IFNα stimulation by rotavirus provides a mechanism for the establishment of the antiviral state extraintestinally by a small amount of virus. Increased levels of serum IFNα have been reported in human pediatric patients infected with rotavirus [43], [44]; levels peaked within two days of symptom onset and disease resolved more quickly in patients with the highest levels of IFNα [43]. Likewise, type I IFN responses were observed in newborn calves infected with bovine rotavirus: animals receiving both high and low doses of viral inoculums generated multiple waves of IFN production, with higher doses correlating with both more rapid IFN responses and a lack of clinical signs [74]. As the ability of type I IFN to activate multiple arms of the innate and adaptive immune response is well documented (reviewed in [75], [76], [77]), and recent data have shown that type I IFN plays a critical role in restricting systemic rotavirus infection [78], we predict that pDC-derived type I IFN plays a significant role in confining rotavirus replication to the intestine while initiating the systemic immune response in the periphery.

In summary, these studies show that primary human pDCs are largely resistant to rotavirus replication, but that they remain responsive and functional following viral exposure, as indicated by cellular activation, maturation and production of multiple cytokines, including TNFα and large amounts of IFNα. The pDC response is demonstrated to require both the viral dsRNA genome and acidification of the pDC endosome, implying the likely initiation of the type I IFN response by TLR7 or TLR9. To our knowledge, these data are the first to show that inactivated rotavirus can efficiently induce a type I IFN response, which differs from what has been seen in fibroblasts and epithelial cells exposed to rotavirus [62]. The restricted ability of rotavirus to replicate in pDCs, combined with the ability of the pDC to effect a type I IFN response in the absence of viral replication, likely serves the purposes of enabling the host to mount an innate antiviral response despite the efficient degradation of the IRFs by rotavirus NSP1 in many host cells. Combined with the novel dispensability of trypsin cleavage of VP4 for the initiation of the pDC response, this creates a mechanism for the establishment of an antiviral state in the systemic extraintestinal environment and likely contributes to the substantial restriction of systemic replication compared to that observed in the intestine [38]. These experiments also provide the first indication that rotavirus infection is capable of locally suppressing IFNα production in primary human cells. Importantly, these findings demonstrate that rotavirus infection of primary human pDCs provides a valuable experimental system for the study of cellular processes, receptors and signaling pathways underlying pDC-mediated antiviral immunity while revealing a potentially novel dsRNA-mediated pathway of IFNα induction.

## Materials and Methods

### Virus preparation and inactivation

Simian (RRV and SA11) tissue-culture adapted rotaviruses were grown in fetal monkey kidney (MA104) cells in the presence of trypsin, except where noted, as previously described [79], [80]. Where indicated, RRV empty and genome-containing TLPs were purified from MA104 cell lysates by cesium chloride density gradient centrifugation following genetron extraction and pelleting through a sucrose cushion, as previously described [81], [82]. DLPs were created by treating TLPs with 20 nM EDTA, thus removing the outer viral proteins VP4 and VP7, as previously described [32]. Purified viral preparations were dialyzed to remove residual cesium chloride prior to pDC stimulus. A portion of the RRV lysate preparation was inactivated by treatment with 40 µg/ml psoralen, followed by exposure to ultraviolet light for 40 minutes, as described [83]. The inactivation of infectivity and transcriptional activity of the RRV in the psoralen preparations was confirmed by cell culture assay and the elimination of endogenous RNA polymerase activity by *in vitro* assay. Except where noted, viral preparations were trypsin activated (5 µg/ml) at 37°C for 20–30 minutes prior to pDC infection. Preparations were endotoxin-free, as determined by *Limulus* amebocyte lysate test (Charles River, Wilmington, MA)**.**

### Virus-like particles

Rotavirus-like particles (VLPs) expressing VP2 and VP6 (2/6 VLPs), or VP2, VP6 and VP7 (2/6/7 VLPs), were generated by coinfecting *Spodoptera frugiperda 9* cells with two or three recombinant baculoviruses, respectively, at a moi ≥5 plaque forming units per cell, as previously described [84]. Briefly, the baculoviruses expressed RF (bovine rotavirus) VP6, RRV VP7, or a fusion protein consisting of GFP fused to the N terminus of RF VP2 deleted in the first 92 amino acids. Infected cultures were collected 5–7 days post infection and purified by cesium chloride density gradient centrifugation. VLPs composed of RF VP2, SA11 Cl3 VP6, SA11 Cl3 VP7 and SA11 4F VP4 (2/4/6/7 VLPs) were similarly prepared, as previously described [85], [86], [87]. The protein concentration of the purified VLP preparations was estimated by the Bradford method.

### Virus and VLP quantification

All viral preparations were titrated by plaque assay on MA104 cells and expressed as plaque forming units per ml, as described [80]. Hemagglutination assays were performed to determine VP4 content of purified genome-containing and empty TLPs, unpurified RRV and 2/4/6/7 VLPs, as described [88], and used to ensure that equal quantities of TLPs were added to pDC preparations. Additionally, DLPs were prepared by treating purified genome-containing TLPs with 20 nM of EDTA, thus removing the outer capsid from the virion and resulting in equal particle counts between the two preparations.

### Isolation of primary human pDCs

PBMCs were isolated from leukoreduction chambers obtained from the Stanford Blood Center (Palo Alto, CA) by centrifugation over ficoll-hypaque (GE Healthcare, Uppsala, Sweden). Plasmacytoid DCs were negatively selected using the pDC Isolation Kit (Miltenyi Biotec, Auburn, CA), according to manufacturer's instructions. To increase the purity of the pDC preparations, consecutive purifications were performed using an AutoMACS (Miltenyi Biotec, Auburn, CA). An average of 2 million pDCs**,** defined as viable, lineage^-^HLA-DR^+^CD11c^-^CD123^+^ cells, were isolated per donor; preparations were routinely >85% pure.

### pDC culture, infection, stimulation and blocking studies

Approximately 2×10^5^ pDCs were exposed to rotavirus particles or 4 µg/mL CpG ODN 2395 (Coley Pharmaceuticals, Ottawa, Canada), as indicated, for 1 hour in serum-free RPMI-1640 (CellGro, Manassas, VA) supplemented with penicillin/streptomycin and L-glutamine (Gibco, Carlsbad CA). pDCs were washed and suspended at a concentration of 1 million pDCs/mL in RPMI-1640 with 10% heat-inactivated fetal bovine serum (Hyclone, Logan, UT), penicillin/streptomycin, L-glutamine and 10 ng/mL IL3 (Peprotech, Rocky Hill, NJ) until harvest. Where indicated, rotavirus infection was blocked using neutralizing monoclonal antibodies to VP5 (mAb 2G4) or VP7 (mAb 159), or as a non-neutralizing control, a VP6 monoclonal antibody (mAb 1E11). In studies determining whether pDC infection was productive, highly-purified pDCs (mean purity: 91.51%) were exposed to RRV at an moi of 10 for one hour; neutralizing mAb 159 was then added for 30 minutes. pDCs were washed 3 times, and were frozen immediately or after overnight culture. The resulting pDC pellets and supernatants were freeze/thawed three times, and virus titer determined by FFU assay, as previously described [89].

### Inhibition of endosomal acidification

Purified pDCs were incubated with 20 nM concanamycin A (Sigma-Aldrich, St Louis, MO) at 37 degrees for one hour prior to infection with live or inactivated RRV (moi 10).

### Flow cytometry (FACS)

Supernatants were harvested by centrifugation of cultured pDCs, which were then washed once with PBS (CellGro, Manassas, VA). The LIVE/DEAD Aqua Dead Cell Stain Kit (Invitrogen, Carlsbad, CA) was utilized to assess cellular viability via amine exclusion. Surface staining was performed using antibodies against human CD3, CD14, CD16, HLA-DR, CD83, CD86, CD123 (BD Biosciences, San Jose, CA), CD11c, CD19, CD20 (eBioscience, San Diego, CA), and BDCA2 (Miltenyi Biotec, Auburn, CA); pDCs were defined as being lineage^-^HLA-DR^+^CD11c^-^CD123^+^. Cellular fixation and permeabilization were performed using Cytofix/Cytoperm (BD Biosciences, San Jose, CA), per the manufacturer's instructions. Following blocking of Fc receptors (Miltenyi Biotec, Auburn, CA), intracellular staining was performed with antibodies to IFNα, total IRF7, phosphorylated IRF7 (pS477/pS479) (BD Biosciences, San Jose, CA)**,** IFNβ (Antigenix America, Huntington Station, NY) or NSP2 (mAb 191). The presence of NSP2 staining of exposed pDCs was considered to be indicative of rotaviral replication in those cells. Direct conjugation of mAb 191 to APC was performed by Chromaprobe Inc (Maryland Heights, MO). Data were acquired using a LSRII and DIVA software (BD Biosciences, San Jose, CA); analysis was performed using FlowJo (Treestar Inc, Ashland, OR).

### Detection of secreted cytokines and chemokines

Supernatants of cultures >85% (mean ± SEM: 90.84%±0.7499) pure for pDCs were analyzed by Luminex using MILLIPLEX MAP (Millipore, Billerica, MA) per the manufacturer's instructions for the presence of IFNα, IFNγ, TNFα, TNFβ, IL1β, IL1RA, IL2, IL4, IL6, IL8, IL10, IL12p40, IL12p70, CXCL10 (IP10), CCL3 (MIP1α), CCL4 (MIP1β), CCL5 (RANTES) and sCD40L. Secreted IFNβ was detected by ELISA, per the manufacturer's instructions (PBL Interferon Source, Piscataway, NJ).

### Statistical analysis

Mann-Whitney and Wilcoxon signed rank tests were performed using GraphPad Prism (GraphPad Software Inc, La Jolla, CA). A p-value of ≤0.05 was considered significant.

## Supporting Information

Figure S1 pDC gating strategy. (A-D) pDCs were defined in these studies as amine^-^ (viable) lineage^-^HLA-DR^+^CD11c^-^CD123^+^ cells. (E) Representative FACS plot demonstrating BDCA2 vs. NSP2 staining of pDCs (red) vs. contaminating HLA-DR- (blue) cells exposed to RRV (n = 12).
(0.39 MB TIF)
