# Supplementary figures and images for: Rotavirus Structural Proteins and dsRNA Are Required for the Human Primary Plasmacytoid Dendritic Cell IFNα Response
Source: PLoS Pathog. 2010 Jun 3;6(6):e1000931. doi: 10.1371/journal.ppat.1000931 (PMC2880586; doi:10.1371/journal.ppat.1000931)

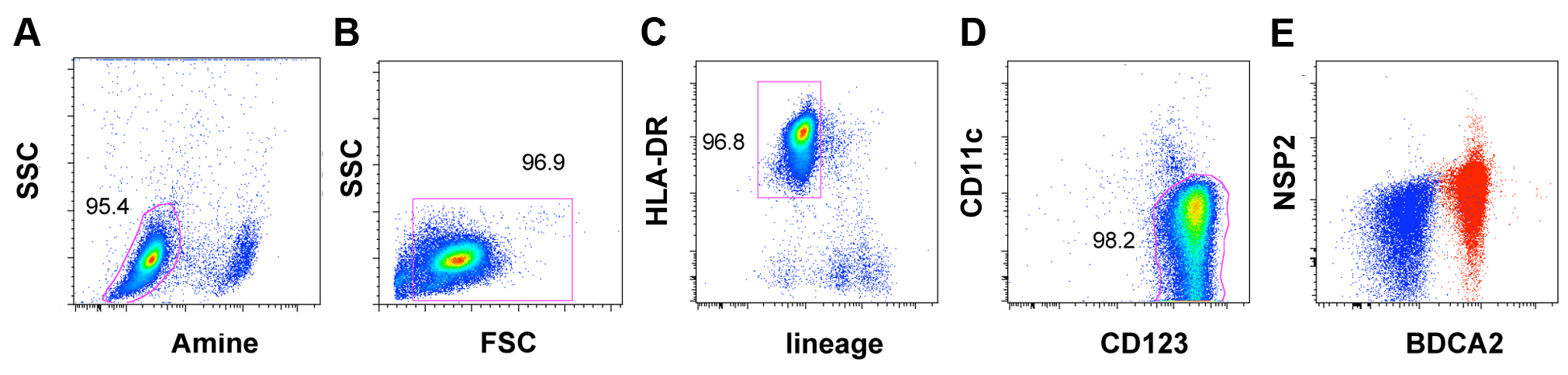

Supplement: Figure S1 — pDC gating strategy. (A-D) pDCs were defined in these studies as amine- (viable) lineage-HLA-DR+CD11c-CD123+ cells. (E) Representative FACS plot demonstrating BDCA2 vs. NSP2 staining of pDCs (red) vs. contaminating HLA-DR- (blue) cells exposed to RRV (n = 12). (0.39 MB TIF) [file ppat.1000931.s001.tif]
